# Platelet-monocyte aggregates: molecular mediators of thromboinflammation

**DOI:** 10.3389/fcvm.2023.960398

**Published:** 2023-05-15

**Authors:** Christina C. Rolling, Tessa J. Barrett, Jeffrey S. Berger

**Affiliations:** ^1^ Department of Medicine, New York University Grossman School of Medicine, New York, NY, United States; ^2^ Department of Oncology and Hematology, University Medical Center Hamburg-Eppendorf, Hamburg, Germany

**Keywords:** monocyte-platelet aggregates, thromboinflammation, antiplatelet therapy, P2Y12 inhibitor, inflammatory diseases, atherosclerosis

## Abstract

Platelets, key facilitators of primary hemostasis and thrombosis, have emerged as crucial cellular mediators of innate immunity and inflammation. Exemplified by their ability to alter the phenotype and function of monocytes, activated platelets bind to circulating monocytes to form monocyte-platelet aggregates (MPA). The platelet-monocyte axis has emerged as a key mechanism connecting thrombosis and inflammation. MPA are elevated across the spectrum of inflammatory and autoimmune disorders, including cardiovascular disease, systemic lupus erythematosus (SLE), and COVID-19, and are positively associated with disease severity. These clinical disorders are all characterized by an increased risk of thromboembolic complications. Intriguingly, monocytes in contact with platelets become proinflammatory and procoagulant, highlighting that this interaction is a central element of thromboinflammation.

## Introduction

Platelets interact and bind to monocytes by a variety of mechanisms, including attachment of platelets to monocytes via platelet P-selectin and monocyte PSGL1, the release of platelet granules containing chemokines and cytokines, and shedding of platelet-derived microvesicles. These interactions result in the upregulation of monocyte proinflammatory surface markers (e.g., CD40), migration (CD11b/CD18), and procoagulant tissue factor (TF), a principal initiator of coagulation. In addition, monocytes exposed to platelets secrete proinflammatory cytokines (TNF-α, MCP-1, IL-1β) and exhibit a proinflammatory transcriptome. Furthermore, platelets skew monocyte and macrophage differentiation towards a proatherosclerotic phenotype.

Our review covers how platelets affect monocytes in inflammatory diseases, and we present recent findings on potential therapeutic strategies to target the platelet-monocyte proinflammatory axis in thromboinflammation.

## Platelets in hemostasis and thrombosis

Derived from megakaryocytes, platelets are small, anucleate cells circulating in the blood for seven to ten days. While crucial to hemostasis and thrombosis, an immunomodulatory effector role for platelets is increasingly apparent (1). Under physiological conditions, circulating platelets become activated when they come in contact with subendothelial collagen following vascular injury. Exposed collagen attaches to von Willebrand factor (VWF) - a large globular multimeric glycoprotein - which then unfolds to a string-like structure. Platelets adhere to tethered VWF via the glycoprotein (GP) Ib-IX-V receptor complex (2) or directly via collagen through the GPVI receptor. Interaction of platelets with collagen and VWF results in platelet activation as shown by changes in the platelet cytoskeleton and release of cytokines, chemokines, and growth factors stored in alpha and dense granules (3). Adenosine diphosphate (ADP) derived from platelet dense granules and thromboxane A2 (TXA2) produced by activated platelets out of arachidonic acid, and other mediators including epinephrine and thrombin generated on the platelet membrane amplify and maintain the initial platelet response by recruiting and activating additional platelets. This ultimately leads to activation of the integrin complex GPIIb/IIIa, a key receptor for platelet adhesion, aggregation, and thrombus stabilization (4). As a result, activated platelets adhere to the vessel wall and form a thrombus, thereby preventing excessive blood loss at the injury site.

In addition to local platelet activation required for thrombosis and wound healing, platelets have effector roles in systemic inflammatory conditions. Platelet activation has been observed in sepsis (5–9), autoimmune disorders (10–14), and chronic proinflammatory conditions, including hyperlipidemia (15–17), atherosclerosis (18–22), and cardiovascular disease (22–26). Resultantly, patients with these diseases express elevated levels of circulating proinflammatory cytokines and chemokines and are also prone to thromboembolic complications (17, 27–29).

## The role of monocytes in thromboinflammation

Monocytes originate in the bone marrow and constitute a subpopulation of approximately 10% of all peripheral blood leukocytes. Monocytes are key mediators of innate immunity. They phagocytose and present antigens, secrete chemokines and cytokines, and can terminally differentiate into different macrophage and dendritic cell subtypes that reside in the extravascular tissues (30, 31).

Based on the expression of surface markers CD14 and CD16, circulating monocytes are traditionally classified as either classical (CD14^++^ CD16^−^), intermediate (CD14^+^ CD16^+^), or nonclassical (CD14^+^ CD16^++^) subpopulations that exhibit different functional properties (32). While classical monocytes are phagocytic, CD16^+^ monocytes are often upregulated in systemic infections, constituting the major cytokine source (33).

The interaction of platelets with monocytes has emerged as a key mechanism connecting thrombosis and inflammation. In support of a proinflammatory and procoagulant platelet effector function, monocyte-platelet aggregates (MPA) are elevated in multiple thromboinflammatory diseases and correlate with disease severity (34). To be able to bind to monocytes, platelets need to become activated (35, 36).

## Platelet activation during inflammation

Inflammatory mediators such as complement factors (37), interleukin-6 (IL-6) (38), IL-8 (39), and tumor necrosis factor-alpha (TNF-α) (40) secreted from activated immune and vascular cells contribute to platelet activation by enhancing their adhesion properties to endothelial cells, elevating collagen-induced aggregation, and enforcing release of TXA_2_.

Under inflammatory conditions, platelet-activating agonists such as TXA_2_, ADP, and thrombin bind to heterotrimeric G protein-coupled receptors (GPCR) expressed on the platelet surface. GPCR consist of different transmembrane spanning G proteins (G_S_, G_i_, G_q_, and G_12/13_) that are associated with platelet receptors including the ADP receptors P2Y_1_ (G_q_) and P2Y_12_ (G_i_), the thrombin receptors PAR1 (G_q_, G_12/13_) and PAR4 (G_q_, G_12/13_), and the TXA_2_ receptor (TP; G_q_, G_12/13_) (41). Upon ligand binding, GPCR induce receptor-specific interconnected signaling pathways that lead to platelet alpha and dense granule release and activation of GPIIb/IIIa (inside-out signaling) (42).

## Pathways of platelet-monocyte interaction in inflammation

Following platelet activation, P-selectin (CD62P) stored in alpha granules translocates to the platelet surface. Platelet P-selectin binds to glycoprotein ligand 1 (PSGL1) which is constitutively expressed on the surface of monocytes. This initial engagement of platelets with monocytes is strengthened by platelet CD40L binding to monocyte CD40, platelet GPVI binding to extracellular matrix metalloprotease inducer (EMMPRIN, CD147) (43) and platelet GPIb attaching to monocyte CD11b/CD18 (MAC-1) (44). Fibrinogen-mediated binding of platelet GPIIb/IIIa to MAC-1 has also been discussed (45) but appears to play only a minor role in the formation of MPA (46). In addition to platelet and monocyte receptor binding, platelets attract monocytes via chemokines and cytokines released from alpha and dense granules, including soluble CD40L (sCD40L) (47, 48), CXCL4 (platelet factor 4) (49), and CCL5 (50). Platelet-derived extracellular vesicles (PEV) including platelet microparticles, microvesicles, and exosomes are tiny circular fragments shed from the platelet membrane, and have been shown to regulate monocyte properties (51–53).

**Figure 1:**
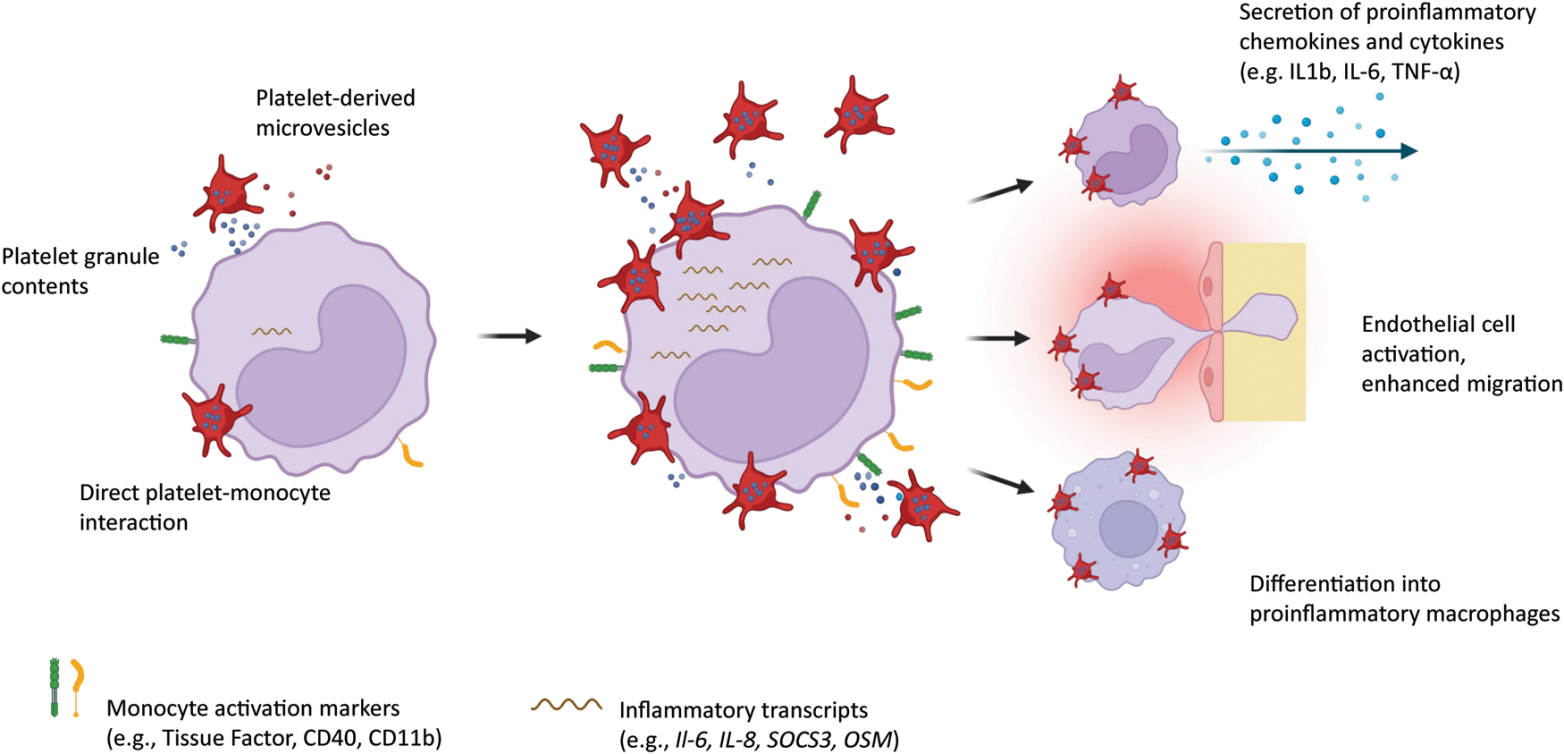
Platelets interact with circulating monocytes by direct attachment onto the monocyte surface, by release of alpha and dense granules containing chemokines and cytokines, and by shedding of microvesicles. Monocytes in turn upregulate inflammation markers on their surface, overexpress proinflammatory transcripts, secrete chemokines and cytokines, show enhanced migration potential, and differentiate into proinflammatory macrophages. Created with BioRender.com

Interaction of platelets with monocytes by direct cell-to-cell contact and platelet-derived mediators facilitates the transition of monocytes to a proinflammatory and procoagulant phenotype. In the following sections, we will outline the different mechanisms by which platelets modify monocytes, and summarize how these mechanisms contribute to inflammation and thrombosis in the clinical setting (Figure 1).

## Activated platelets modify the phenotype of circulating monocytes

Monocyte subtypes as defined by their expression of surface markers CD14 and CD16 can be modified by platelet interaction: Platelets appear to preferentially bind to CD16-bearing monocytes (16, 54). When bound to platelets, classical (CD14^++^CD16^−^) human monocytes upregulate surface CD16 - likely via platelet-derived transforming growth factor-β (TGF-β) inducing cyclooxygenase 2 (COX-2) upregulation and prostaglandin E2 synthesis in monocytes (55–57). High levels of antigen presenting-related molecules and secretion of proinflammatory cytokines and chemokines characterize these monocyte populations. In support of a proinflammatory platelet effector role, intermediate monocytes are expanded in various inflammatory disorders (34). Several other monocyte activation markers linked to atherosclerosis and inflammation are higher expressed on monocytes attached to platelets than monocytes without adhered platelets, including CD11b/CD18, CD40, PSGL1, HLA-DR, CD86, CD54, and CCR2 (34, 58).

Findings from several studies support a platelet-mediated proinflammatory monocyte phenotype (59). For example, activated platelets adhering to monocytes via the P-selectin PSGL1 axis in combination with alpha granule-released, CD40L, CXCL4 and CCL5 induce expression and secretion of monocyte chemoattractant protein-1 (MCP), IL-8, TNF-α, IL-1β, and IL-6 (50). Another proinflammatory mediator secreted by activated platelets is beta (β)-2 microglobulin which has been shown to induce CD16+ monocytes and augment monocyte inflammatory cytokine secretion (60, 61). β-2 microglobulin is also a component of the physiologic plasma compartment. Hence, the composition and interplay of platelet granule-derived and plasma molecules within the close monocyte-platelet interaction may shape the monocyte outcome rather than single molecular mediators. For example, β2 microglobulin and platelet TGF-β exert their effects on the same monocyte receptor but through different downstream signaling cascades, leading to different (proinflammatory vs. pro-reparative) monocyte phenotypes (61).

Intriguingly, monocytes attached to platelets have increased TF expression on their surface (62), resulting in thrombin-mediated fibrin generation and clot formation (63). Under physiologic conditions, TF is expressed on monocytes in an encrypted (inactive) form but can be decrypted (activated) into its procoagulant isoform upon proinflammatory stimuli (64). Activated platelets induced rapid TF upregulation on monocytes (65). This is mediated by platelet-bound P-selectin and does not require *de novo* protein synthesis (65) albeit incubation of monocytes with soluble P-selectin or platelets over several hours has been shown to induce TF (*F3*) gene expression (66). Additionally, soluble P-selectin shed from activated platelets contributes to the formation of procoagulant MV (67), emphasizing the contribution of platelets to coagulation activation.

Exposure of circulating monocytes to platelets also affects and modulates their subsequent differentiation into tissue-resident macrophages and dendritic cells: For example, platelets - via direct P-selectin-PSGL1-mediated cell-cell interaction - induced maturation of monocytes into antigen-presenting dendritic cells (68). In a murine sepsis model, activated platelets polarized monocytes toward proinflammatory M1 macrophages (69, 70). This reprogramming was initiated as soon as platelets attached to the monocyte surface and could not be altered later on during the differentiation progress (69). Hence, depending on the underlying pathogenesis, platelets shape the monocyte effector function in circulation and define the fate of macrophage recruitment into extravascular tissues. Platelet-mediated macrophage polarization plays an important role in chronic inflammatory diseases such as atherosclerosis and will be outlined further in the upcoming section.

In summary, activated platelets are required to accelerate monocyte-driven inflammation and thrombosis, two tightly interconnected pathogenic mechanisms that profoundly impact on cardiovascular disease (CVD) and beyond.

## Monocyte-platelet interaction in atherosclerosis and cardiovascular disease

Atherosclerosis is the underlying pathology in most cases of CVD including myocardial infarction (MI), stroke, and peripheral artery disease (PAD) (71). Atherosclerosis is a chronic non-resolving inflammatory disease characterized by the formation of lipid-rich calcified plaques within the arterial wall of large and medium-sized vessels due to transmigration and accumulation of activated proinflammatory immune cells consisting of macrophages and T cells. Rupture or erosion of an unstable atherosclerotic plaque results in life-threatening thromboembolic events.

While low MPA levels of around 5%–20% are a normal phenomenon observed in healthy populations (54, 72), their rise is indicative of inflammatory processes and is associated with atherothrombotic complications: Patients with PAD and CVD and/or cardiovascular risk factors, including diabetes mellitus (DM), arterial hypertension and hyperlipidemia exhibit elevated levels of circulating MPA (36, 47, 54, 73–75). Elevated levels of MPA in patients with acute MI were further increased in patients that developed in-hospital adverse events (74).

Circulating monocytes in CVD and other chronic inflammatory disorders are characterized by elevated expression of CD40, a member of the TNF receptor superfamily (76). Proinflammatory cytokines including IFN-γ, IL-1, and TNF-α induce upregulation of monocyte CD40, and monocytes attached to platelets have higher CD40 expression on their surface (34, 77). Importantly, CD40 is a receptor for CD40L which is on the surface of and a marker for activated platelets (48). CD40L-CD40 interaction does not only stabilize MPA but also enhances monocyte migration into the arterial wall (19, 78). CD40-activated macrophages then secrete inflammatory cytokines and matrix metalloproteinases, thereby contributing to plaque destabilization and rupture. In a murine atherosclerosis model, CD40 deficient mice had lower MPA and platelet-mediated leukocyte-endothelium interactions resulting in decreased plaque formation (19). In response to CD40 binding, platelet CD40L is shed from activated platelets into the circulation, which further augments platelet activation (79–81). Notably, platelet-derived CD40L contributes to P-selectin-mediated TF upregulation on monocytes (82, 83). TF expressing macrophages, as well as elevated levels of circulating TF, have been observed in patients with atherosclerosis (84, 85), metabolic cardiovascular risk factors (86–88), and in acute coronary syndrome (89–91).

CXCL4 is one of the most abundant chemokines released from alpha granules upon platelet activation (92). Importantly, CXCL4 has been detected in human carotid atherosclerotic plaques and positively correlates with lesion grade and presence of clinical symptoms (19, 93). The pathogenic role of CXCL4 in accelerating atherosclerosis is mediated, in part, by augmenting the arrest of monocytes on endothelial cells in conjunction with CCL5 (RANTES) (94).

Platelets not only induce attachment and facilitate transmigration of monocytes into the subendothelial space (95) and into atherosclerotic lesions (20) but also shape the subsequent macrophage phenotype: Monocytes exposed to platelet-derived CXCL4 differentiate into proinflammatory M4-type macrophages, which have been suggested as crucial contributors to plaque rupture in coronary artery disease (96). *In vitro* studies and murine models of atherosclerosis demonstrated that oxidized low-density lipoprotein (oxLDL) generated under chronic inflammatory conditions promoted platelet-monocyte interaction with subsequent monocyte extravasation and foam cell formation (16). Foam cells are lipid-laden macrophages that are a major constituent of atherosclerotic plaques (97).

Findings from our group confirm that platelets drive atherogenesis by inducing proinflammatory monocytes and macrophages: We reported that platelet competent atherogenic *Ldlr*^−/−^ mice had increased monocyte surface expression of the adhesion receptor CD11b and had higher expression of inflammatory transcripts *CCL2*, *IL6*, and *CD11b* relative to platelet-depleted mice. Furthermore, in the presence of platelets, plaque macrophages were skewed towards a proinflammatory phenotype as defined by upregulation of *SOCS3,* which promoted proinflammatory cytokine production of IL-6, IL-1b, and TNF-α (20). Consistently, *SOCS3* mRNA expression of whole blood correlated with platelet activity and MPA formation in a clinical dataset of women with MI and in patients with symptomatic lower extremity atherosclerosis (20).

Platelets have been shown to also activate dendritic cells (DC) via the P-selectin-PSGL1 axis, thereby contributing to atherosclerosis progression in hyperlipidemic mice (98). This is mediated by toll-like receptor 4 (TLR4) signaling pathways, leading to enhanced secretion of inflammatory cytokines, T cell communication, and adhesion and migration properties of platelet-bearing DC (98).

In addition to physical contact and secretion of granule contents, platelets can modulate monocyte and macrophage responses by shedding of PEV into the circulation. There is evidence that PEV (via P-selectin) adheres to monocytes, thereby inducing cytokine and TF production, and contributing to atherothrombosis in CVD (99). Interestingly, P-selectin-positive and fibrinogen-positive PEV were elevated in patients six months after acute MI (100). Procoagulant PEV may also contribute to monocyte-endothelial interaction, thereby promoting atherosclerosis initiation and progression (101, 102).

Genetic RNA sequencing of platelets from CVD patients allows the identification of new mediators in MPA formation and monocyte reprogramming: Platelets from patients with symptomatic PAD were enriched with *myeloid-related protein-14* (*MRP-14*) mRNA and protein. MRP-14 augmented the expression of P-selectin, thereby enhancing MPA (21). Elevated serum levels of MRP14 were found in PAD patients with incident cardiovascular and limb events, underscoring a clinically important role for this protein (21).

## Monocyte-platelet interaction in autoimmune disorders

Patients with autoimmune diseases such as Systemic lupus erythematosus (SLE) and rheumatoid arthritis (RA) are at higher risk of developing atherosclerotic and thromboembolic complications (103, 104). Again, elevated levels of MPA in patients with vasculitis, rheumatoid arthritis, and SLE highlight the tight interconnection between inflammation and atherothrombosis (58, 105–108). Monocytes attached to platelets exhibited higher levels of proinflammatory CD54, CD16, CD86, and HLA-DR in SLE patients (58). Additionally, procoagulant microvesicles shed from activated platelets have been implicated in inducing proinflammatory monocytes in RA and SLE (51, 106, 109).

In RA patients, activated platelets attaching to intermediate CD16+ monocytes contributed to elevated MPA formation and inflammatory cytokine secretion via CD147 signaling (106). High platelet activation and platelet-leukocyte aggregation were associated with enhanced TF-dependent global coagulation activation in SLE patients (108).

## Monocyte-platelet interaction in infection and inflammation

In addition to a hyperreactive platelet phenotype and a heightened incidence of thromboembolic complications, elevated levels of circulating MPA were a prominent clinical finding in patients with severe COVID-19 (110–113). Platelet-monocyte interaction in COVID-19 was strongly associated with monocyte TF expression and global coagulation activation as shown by elevated fibrinogen and D-dimers (112). Interestingly, upregulation of P-selectin-dependent monocyte TF expression could be reproduced when monocytes from healthy donors were co-cultured with platelets from COVID-19 patients, once more underlining the proinflammatory effector role for platelets in COVID-19 (112)_._

In dengue virus infection, platelets are activated - evidenced by increased P-selectin and release of CD40L and cytokines- upon direct contact with the virus. As a consequence, elevated MPA can accelerate the monocyte inflammatory response (114–116). In an experimental cerebral malaria model, platelet-derived CXCL4 drove monocyte cytokine production in *Plasmodium berghei* infected mice (117).

Sepsis is a life-threatening organ dysfunction caused by a dysregulated host response to infection (118) and is characterized by platelet activation and platelet-monocyte interaction (119). Elevated levels of circulating MPA were associated with higher morbidity and mortality and correlated with thromboembolic complications in patients with sepsis (120, 121).

While the majority of studies clearly show a proinflammatory role for MPA, platelets exhibited anti-inflammatory properties on the macrophage phenotype in a murine endotoxemia model of severe septic shock (122). These seemingly divergent findings indicate that the effector role of platelets is complex and depends on the underlying clinical disease and severity. More studies are warranted to delineate and better understand the divergent functions of MPA.

## Interaction of platelets with neutrophils and lymphocytes

In addition to monocyte-platelet interaction, formation of neutrophil-platelet aggregates (NPA) represents another intercellular connection linking thrombosis and inflammation. Similar to MPA, elevated circulating NPA have been observed in CVD (123), PAD (124), in COVID-19 (125), and have been linked to an enhanced risk of deep venous thrombosis (126). Platelets – via the P-selectin-PSGL1 axis – activate and induce neutrophils to release neutrophil-extracellular traps (NETs) into circulation (127). NETs consist of DNA, histones, and neutrophile-derived enzymes, most importantly myeloperoxidase, and constitute the most crucial platelet-mediated effect on neutrophils to promote thrombosis in inflammation (128). Since NPA and MPA are found to be simultaneously elevated in proinflammatory conditions (129), activated platelet induce a prothrombogenic state via both leukocyte subpopulations by enhancing their respective inflammatory response *in vivo*. In contrast, while the magnitude of circulating lymphocyte-platelet aggregates does not change in inflammatory, thrombotic, and atherosclerotic diseases, platelet-lymphocyte interaction has been delineated to exert a specific role in cancer: For example, platelets attenuated T cell activity in cancer patients ex vivo (130), and promoted tumor progression via suppression of CD8 T cells in murine cancer models (131).

In summary, platelets differentially modulate leukocyte activity in response to the underlying disease, with MPA and NPA being important contributors in thromboinflammatory diseases.

## Can platelet inhibitors prevent MPA formation?

Targeting MPA has come into focus as an interesting therapeutic strategy. Interrupting the P-selectin-PSGL1-axis offers therapeutic potential in preventing (athero)thrombosis as shown in preclinical models (132–134). Importantly, since P-selectin is also present on activated endothelial cells (135), using an anti-P-selectin antibody allows to attenuate microvascular inflammation in addition to preventing proinflammatory platelet-monocyte interaction.

However, the synthetic P-selectin inhibitor PSI-697 failed to decrease MPA in healthy smokers (136). So far, among P-selectin inhibitors, only crizanlizumab was approved in 2019 (137) and received a conditional marketing authorization by the European Medicines Agency (EMA) in 2020 (138) for the prevention of pain crises in sickle cell disease. In this clinical setting, crizanlizumab is used to prevent sickle erythrocytes to adhere to and activate P-selectin expressing endothelial cells (139). Due to unpublished results of the STAND trial, fate is currently unclear (140).

Recently, a phase 2 clinical trial investigated the effects of crizanlizumab on patients hospitalized with moderate COVID-19 which is associated with endothelial dysfunction and vascular inflammation. Here, crizanlizumab therapy was associated with significantly decreased plasma levels of prothrombin fragments and thrombin-antithrombin complexes, both laboratory markers for coagulation activation (141). Subsequently, the effectiveness of crizanlizumab in preventing adverse clinical outcomes in hospitalized COVID-19 patients is being investigated in a large international phase 4 randomized clinical trial (142).

While PSGL1 and P-selectin inhibitors directly interfere with platelet-monocyte interaction as well as with leukocyte and erythrocyte attachment onto endothelial cells, most therapeutics that are in clinical use block platelet signaling pathways upstream of the release of alpha granule contents like P-selectin. Targeting the ADP-activated P2Y_12_ pathway effectively reduces MPA in several clinical studies: In patients with acute MI, atherosclerotic vascular disease, and cardiovascular risk factors including diabetes mellitus, intake of P2Y_12_ inhibitor therapy was associated with MPA reduction *in vivo* and ex vivo (73, 75, 143, 144). Importantly, the P2Y_12_ inhibitor ticagrelor resulted in reduced MPA and dampened myocardial inflammation (as shown by increased FGD-uptake and lower cardiac ejection fraction) in acute MI patients (75). Moreover, P2Y_12_ blockade reduced monocyte TF expression (145, 146) and attenuated circulating levels of IL-8, TNF-α, and CCL2 in an experimental human model of systemic inflammation (147)_._ In patients with acute coronary syndrome, the P2Y_12_ inhibitor clopidogrel diminished circulating levels of proinflammatory TNF-α and C-reactive protein (144). Of note, pneumonia patients had significantly reduced plasma IL-6 under ticagrelor compared to patients taking placebo and needed less supplemental oxygen (148). In contrast, in non-critically ill patients with COVID-19, P2Y_12_ blockade in addition to heparin compared to heparin alone did not result in increased odds of improvement in organ support-free days (142) and – equally to COX-1 inhibitors - only had a low likelihood of improving the number of organ support-free days in critically-ill COVID-19 patients when compared to patients without platelet inhibitors (149). Accordingly, depending on the underlying inflammatory condition, the effects of platelet inhibitors on defined clinical outcomes may vary.

COX-1 inhibition with aspirin did not lower MPA assessed *in vitro* and following 1-week of low-dose aspirin in healthy volunteers ex vivo (54). This might be due to aspirin not directly interfering with GPCR signaling-induced alpha degranulation and P-selectin expression. While aspirin therapy was insufficient to decrease MPA in acute stroke patients, it attenuated MPA ex vivo when whole blood from healthy volunteers was stimulated with thrombin receptor activator peptide 6 (TRAP-6) (150). Different outcomes of MPA under aspirin seem to depend on the measurement methods. Aspirin did not reduce mortality linked to sepsis in healthy elderly patients (151). Importantly, in a murine model of severe bacteremia, aspirin even enhanced inflammation and was associated with higher mortality (122), indicating a potential detrimental effect in severe sepsis.

Clinical data on the effect of PAR1 inhibitors on platelet-monocyte interaction are scarce. PAR1 inhibition attenuated MPA formation in whole blood stimulated with the TXA_2_ analog U-46619, and consequently, monocyte attached to platelets had less surface expression of CD40 and TF (129). In another in-vitro analysis, incubation of whole blood from healthy donors with PAR1 inhibitors prevented TRAP-6 - but not PAR4 - or collagen-related peptide (CRP)-induced MPA formation, emphasizing that the efficacy of platelet inhibitors is dependent on the activation pathway (152). There is conflicting data on the effect of GPIIb/IIIa inhibitors on MPA with some studies even showing paradoxically elevated MPA levels under eptifibatide (153, 154).

In addition to blocking cell surface receptors, platelet activation can also be targeted by intracellular inhibition of phosphodiesterases (PDE). As a result, intracellular levels of cyclic adenosine monophosphate (cAMP) and/or cyclic guanosine monophosphate (cGMP) accumulate, which eventually disrupts the rearrangement of the actin cytoskeleton required for platelet activation and granule release (155). While the PDE inhibitor dipyridamole did not attenuate thrombin- or collagen-stimulated monocyte-platelet aggregates *in vitro* (156), patients under combined treatment with aspirin and dipyridamole following a transitory ischemic attack (TIA) had lower MPA when exhibiting dipyridamole responsiveness in a platelet function test in contrast to patients that were identified as dipyridamole non-responders (157).

Platelet GPIb and its ligand MAC-1 on leukocytes are newer potentially therapeutic targets (158). Interestingly, inhibiting this interaction resulted in impaired thrombus formation and delayed thrombosis in murine models (158). In a phase I study on healthy volunteers, GPIb-binding anfibatide, a snaclet (snake C-type lectins) purified from snake venom, showed antithrombotic efficacy without affecting haemostasis (159). Clinical trials are anticipated to investigate the clinical efficacy of GPIb and MAC-1 inhibitors in attenuating thrombosis linked to inflammation.

In conclusion, targeting platelet-monocyte interactions is likely to be beneficial to various inflammatory diseases. Among platelet inhibitors, directly targeting platelet P-selectin or preventing its release from alpha granules by upstream blockade of P2Y_12_ have been shown to be the most potent strategies to attenuate MPA. Further clinical studies would be welcome to evaluate the effectiveness of antiplatelet therapy-mediated MPA decrease on clinical outcomes within the different inflammatory diseases.

**Figure 2:**
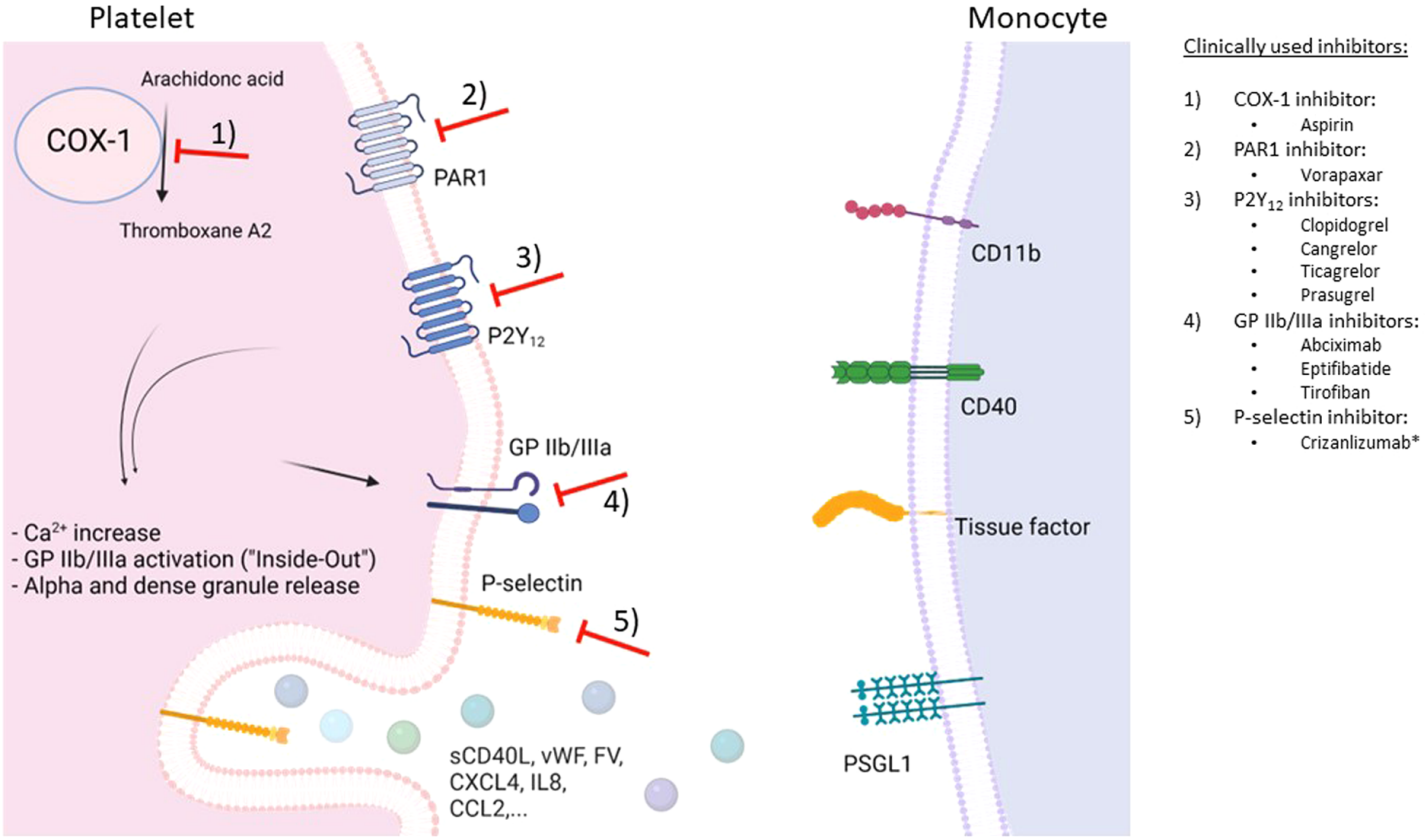
Schematic overview of potential therapeutic targets to prevent monocyte-platelet interactions. Depicted are G protein-coupled receptors (GPCR) associated with platelet receptors P2Y_12_ and PAR1 as well as GPIIb/IIIa receptors on the platelet surface, and cyclooxygenase-1 (COX-1)-mediated production of platelet-activating thromboxane A2. Following platelet activation, many interconnected pathways result in intracellular calcium increase, activation of GPIIb/IIIa, and release of alpha and dense granule contents. P-selectin (released from alpha granules) translocates to the platelet surface and binds to P-selectin glycoprotein ligand-1 (PSGL1) on the monocyte surface, resulting in monocyte-platelet aggregates (MPA). In turn, monocytes become proinflammatory and procoagulant. *has received a conditional marketing authorization by the European Medicines Agency in 2020. Figure created with BioRender.com

Potential therapies targeting MPA are summarized in (Figure 2).

**Figure 3:**
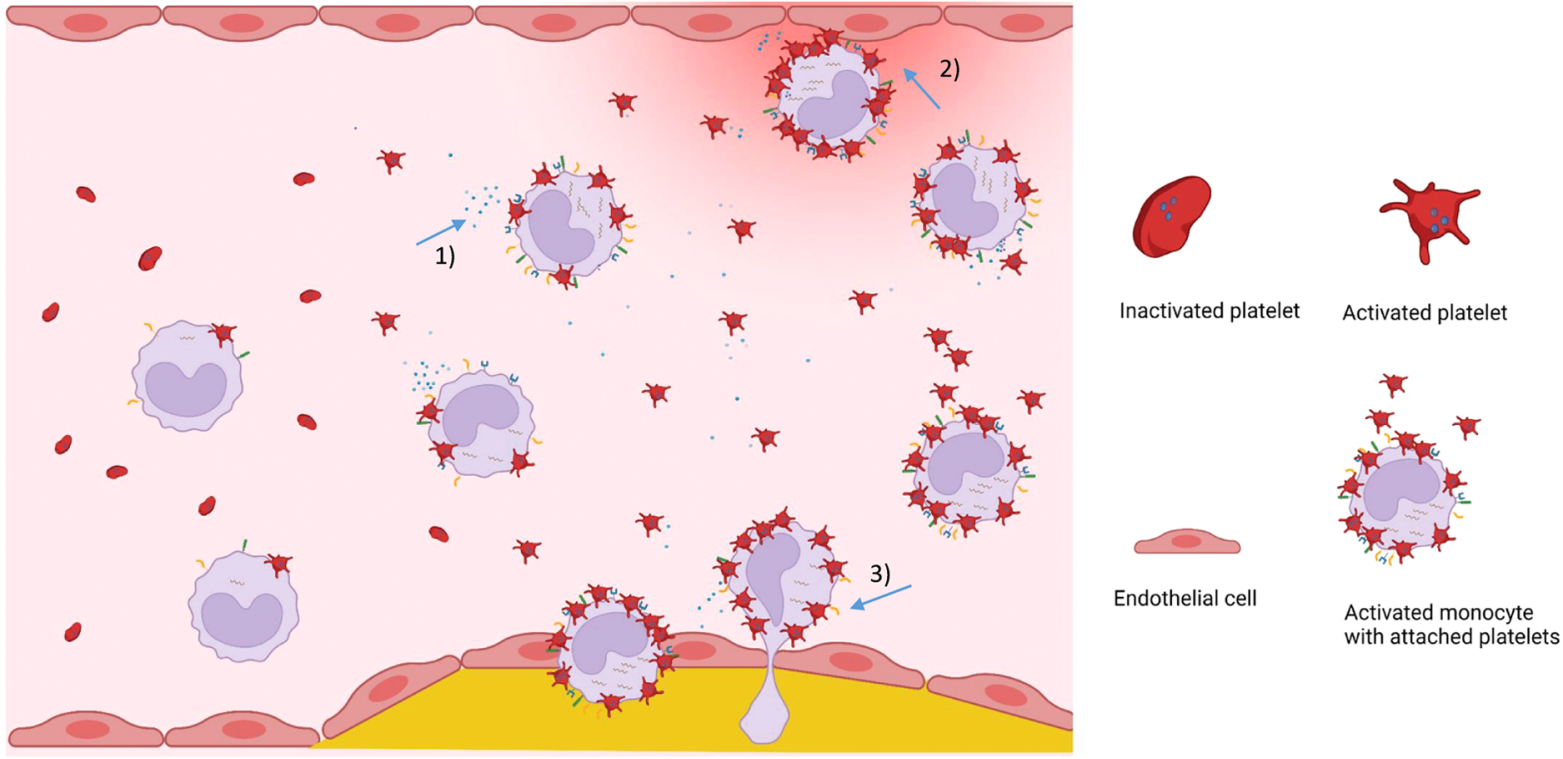
Visual summary on the role of MPA in cardiovascular disease. Plasma levels of circulating monocyte-platelet aggregates are increased in inflammatory diseases. Platelet-induced proinflammatory monocytes secrete inflammatory cytokines and chemokines (1), attach to and activate endothelial cells (2), show enhanced transendothelial migration potential (3), and differentiate into proinflammatory macrophages that contribute to the progression of atherosclerosis. Figure created with BioRender.com

## Measurement of monocyte-platelet interactions

Measuring monocyte-platelet aggregates in whole blood by flow cytometry is a straightforward and commonly used method to assess ex vivo markers of thromboinflammation in clinical studies. However, since there is no clear consensus on the best methodological approach, various preanalytical variables such as blood drawing (venous puncture or access via central venous catheter), sample handling (e.g., temperature, transportation, storage), and processing (centrifugation configuration) may lead to incongruent results (72, 160). When interpreting MPA data, it is therefore important to know the experimental set-up and techniques applied for analysis. The following cornerstones need to be considered when measuring MPA by flow cytometry: To avoid any artificial platelet activation, blood collection should always be performed by direct venous puncture without a tourniquet and after an initial discard. The type of anticoagulant (e.g., heparin, EDTA, or sodium citrate) in the collection tube may affect the magnitude of monocyte-platelet aggregate levels (160). Following staining with monocyte and platelet antibodies, blood can be fixed, lysed and stored at 4°C for up to 24 h (160). Monocytes are identified by forward and side scatter properties and by CD14 (and CD16) expression. For platelet labeling, antibodies binding to constitutively expressed platelet surface glycoproteins, e.g., CD41, CD42a, CD42b, or CD61, can be used. MPA are quantified as the percentage of monocytes positive of a platelet marker within the gated monocyte population. Alternatively, multispectral imaging flow cytometry combining flow cytometry and imaging data provides additional information on platelet binding to individual monocytes (161).

## Conclusions

Accumulating evidence demonstrates that activated platelets induce a proinflammatory monocyte phenotype, affecting the inflammatory response in acute and chronic inflammation. Mediated by direct platelet-monocyte interactions and platelet-derived cytokines, chemokines, and shedding of procoagulant extracellular vesicles. As a consequence, monocytes release inflammatory cytokines into circulation, become procoagulant, and differentiate into proatherogenic macrophages (Figure 3). Targeting platelet effector cell properties – and hence monocyte-driven inflammation and coagulation – appears to be a promising strategy in inflammatory settings beyond CVD.
